# Neonatal Death and Heart Failure in Mouse with Transgenic HSP60 Expression

**DOI:** 10.1155/2015/539805

**Published:** 2015-10-04

**Authors:** Tsung-Hsien Chen, Shan-Wen Liu, Mei-Ru Chen, Kuan-Hung Cho, Tzu-Yin Chen, Pao-Hsien Chu, Yu-Ying Kao, Ching-Han Hsu, Kurt Ming-Chao Lin

**Affiliations:** ^1^Institute of Biomedical Engineering and Nanomedicine, National Health Research Institutes, Zhunan, Miaoli County, Taiwan; ^2^Department of Biomedical Engineering and Environmental Sciences, National Tsing Hua University, Hsinchu, Taiwan; ^3^Division of Cardiology, Department of Internal Medicine, Chang Gung Memorial Hospital, College of Medicine, Chang Gung University, Taipei, Taiwan; ^4^Department of Biotechnology, Chia Nan University of Pharmacy and Science, Tainan, Taiwan

## Abstract

Mitochondrial heat shock proteins, such as HSP60, are chaperones responsible for the folding, transport, and quality control of mitochondrial matrix proteins and are essential for maintaining life. Both prosurvival and proapoptotic roles have been proposed for HSP60, and HSP60 is reportedly involved in the initiation of autoimmune, metabolic, and cardiovascular diseases. The role of HSP60 in pathogenesis of these diseases remains unclear, partly because of the lack of mouse models expressing HSP60. In this study we generated HSP60 conditional transgenic mice suitable for investigating *in vivo* outcomes by expressing HSP60 at the targeted organ in disease models. Ubiquitous HSP60 induction in the embryonic stage caused neonatal death in mice at postnatal day 1. A high incidence of atrial septal defects was observed in HSP60-expressing mice, with increased apoptosis and myocyte degeneration that possibly contributed to massive hemorrhage and sponge-like cardiac muscles. Our results showed that neonatal heart failure through HSP60 induction likely involves developmental defects and excessive apoptosis. The conditional HSP60 mouse model is useful for studying crucial biological questions concerning HSP60.

## 1. Introduction

Heat shock proteins (HSPs) are molecular chaperones responsible for critical functions, such as protein folding, transport, and signaling. Under stress, HSPs preserve proteins in their native forms and refold denatured proteins [1]. Mitochondrial chaperonins, such as HSP60, together with HSP10, refold all proteins imported into the mitochondrial matrix and facilitate the restoration of unfolded or misfolded matrix proteins under normal and stressed environments; hence, they are vital for maintaining mitochondrial functions, including ATP synthesis [2–4]. Although the fundamental biology of HSP60 particularly on mechanisms of substrate folding has been explored extensively, HSP60-related studies are gaining increasing attention because new findings about the roles of mitochondrial, cytosolic, and extracellular HSP60 in a wide array of diseases have emerged [5]. Most HSPs are prosurvival, although both prosurvival and prodeath roles of HSP60 have been reported. The protective role of HSP60 in various cell types, including cardiac myocytes, has been reported [6–13]. Preservation of mitochondrial capacity by HSP60 with reduced cytochrome C release during stress challenges and subsequent apoptosis reduction were among the common findings of the protection by HSP60. Upregulated HSP60 expression in several studies suggested the prosurvival and antiapoptotic roles of HSP60 in cancers [14], such as cervical [15], prostate [16], and colorectal cancers [17] and Hodgkin lymphoma [18]. The proapoptotic role of HSP60 was documented in muscles [19] and in various cancer cell lines [20, 21]; for example, malignancy of esophageal squamous cell carcinoma [22] and ovarian [23] and bladder cancers [24] was found inversely correlated with HSP60 expression. The findings of HSP60 accumulation in cytoplasm in various apoptosis models and HSP60 binding to procaspase-3 supported the proapoptotic properties of HSP60 [21, 25]. Besides the discrepancy among reports of* in vitro* studies, the roles of HSP60 in physiology and pathology cannot be fully elucidated without using transgenic (Tg) mouse models expressing HSP60* in vivo*.

The role of extracellular HSP60 in viral infection, innate or adaptive immunity, and atherosclerosis has been widely documented [26–28]. Hepatitis B virus replication induced HSP60 production, and HSP60 has a critical role in regulatory T cells functions, particularly in IL-10 secretion [28]. Innate immune cell surface receptors, namely, TLR2, TLR4, and CD14, are known receptors for extracellular HSP60, resulting in the release of mediators such as TNF-*α*, IL-1*β*, IL-6, and NO, which in turn enhance inflammation in type 1 diabetes mellitus (DM), atherosclerosis, arthritis, and transplant rejection.

HSP60 expression in the heart is reduced in ageing and metabolic diseases [29]. Caloric restriction that is shown increases lifespan restored aging-related decline of HSP60 expression in the heart and improved cardiovascular functions [29]. The reduction in HSP60 level contributing to low insulin sensitivity and to dysfunctions in metabolic syndrome and type 2 DM was proposed [30]. Because of the importance of HSP60 in many diseases, Tg mouse models with inducible and tissue-specific HSP60 expression can provide useful insights and expand the current understanding of these diseases.

Previously nonobese diabetic (NOD) HIIE*α*-HSP60 Tg mice, in which HSP60 is driven by the MHC class II-E*α* (HIIE*α*) promoter, achieved HSP60 expression specifically in the thymus and bone marrow and suppressed susceptibility to autoimmunity induced DM [31]. Tg mice expressing truncated HSP60 instead of the entire HSP60 were also reported, and the resultant cytosolic HSP60 Tg mice were resistant to hepatic stress with increased cell survival [32]. However, Tg mice with HSP60 expression in other tissues have not been reported. Attempts to generate a conventional HSP60 mouse model did not succeed because Tg founders as chimeras failed to survive (personal communications). In this report, we present a viable and healthy inducible HSP60 Tg mouse (G-Lox-HSP60) in FVB strain driven by a ubiquitous CMV early enhancer/chicken *β*-actin promoter (CAGGS). This model overcame the early lethality of the conventional Tg approach. After crossing the G-Lox-HSP60 mouse with the Cre mouse, we observed neonatal death in mice with HSP60 expression from the embryo stage.

## 2. Materials and Methods

### 2.1. Generation of G-Lox-Hsp60 and EGFP-Cre Transgenic Mice

**Figure 1:**
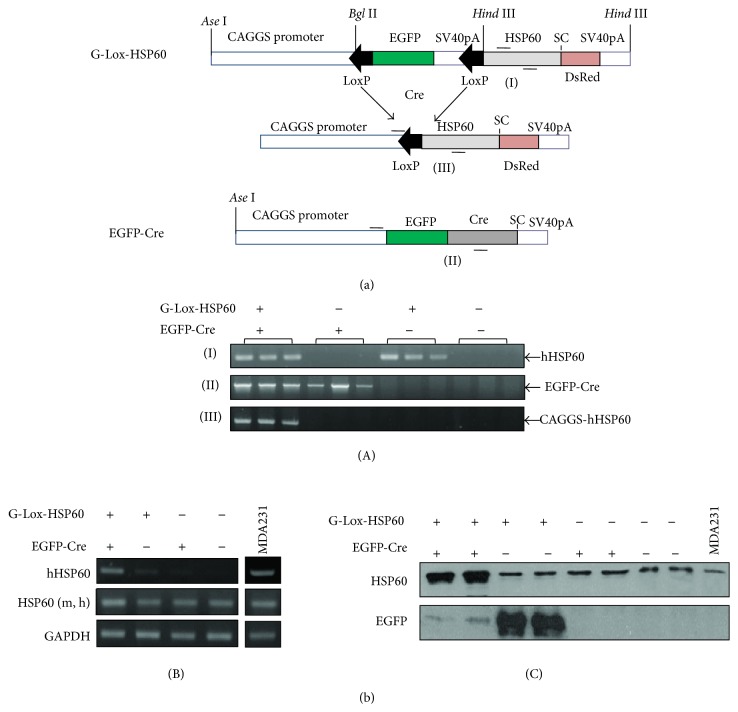
Generation of conditional human HSP60 transgenic mice. (a) G-Lox-HSP60 and EGFP-Cre Tg vectors. The CAGGS promoter was used to drive both Tg vectors. Placed between two LoxP sites, the GFP cDNA with 3 stop codons (SC) and the SV40 polyA sequence was used to inhibit the expression of the downstream transgene. After the second LoxP site, a full-length human HSP60 cDNA with endogenous SC and a portion of the last exon was inserted in front of ATG of the DsRedT1 cDNA sequence. After the LoxP sites were rejoined using the Cre DNA recombinase, the HSP60/DsRedT1 transcript was expressed; however, only HSP60 translated into proteins. (b) (A) Analysis of four possible littermate genotypes using PCR on tail DNA. (I) PCR amplification using the primer pair complementary to human HSP60 but not to mouse HSP60, (II) amplification of EGFP-Cre, and (III) the reaction to identify double transgenic (H^+^/C^+^) mice by amplifying the abridged sequence from CAGGS promoter to human HSP60. (B) RT-PCR results of human HSP60 mRNA expression in the neonatal mouse heart. HSP60 (m, h) indicates the PCR reaction to amplify HSP60 mRNA of both mouse and human origions. GAPDH amplification served as the internal control. MDA-MB231 human breast tumor cells mRNA were used as the positive control for human HSP60. (C) Western blotting for HSP60 and EGFP proteins in neonatal hearts. Anti-HSP60 antibodies recognize both mouse and human HSP60.

A G-Lox-Hsp60 mouse, FVB/N-Tg(ACTB-EGFP/HSPD1)14Klin, was generated by using the Tg vector (Figure 1(a)) and constructed by inserting the following fragments: CAGGS promoter, 2 LoxP sites at the start and the end separated by an EGFP coding sequence and an SV40 polyA sequence (pEGFP-C2, Clontech, Mountain View, CA), and a human HSP60 cDNA (NM_002156, 102–1920 bp) inserted into the pDsRed-N1 vector (Clontech), in which the CMV enhancer was removed. An* Apa*LI-*Afl*II fragment was used for microinjection into the FVB blastocysts. EGFP-Cre mouse, FVB/N-Tg(ACTB-EGFP.CRE)21Klin, was constructed by inserting CAGGS and Cre (AF334827, 1767–2789 bp) into the pEGFP-C2 vector, in which the CMV enhancer was removed [33]. For unknown reasons, the green fluorescence of the EGFP-Cre mouse was extremely weak. G-Lox-DsRed mice have been described previously [34]. All animal experiments were conducted in accordance with the accepted standards of animal care and were approved by the Institutional Animal Care and Use Committee of the National Health Research Institutes, Taiwan. The following PCR primers pairs were used for genotyping for the results in Figure 1(b)(A): (I) 5′-AATGCTCACCGTAAGCCTTT-3′, 5′-CCATCTGAAAGTTTTGCAAG-3′, *T*_*m*_ = 65.5°C, 407 bp; (II) 5′-CTGCTAACCATGTTCATGCC-3′, 5′-ACCGTCAGTACGTGAGATATCTT-3′, *T*_*m*_ = 58°C, 1443 bp; and (III) 5′-CTGCTAACCATGTTCATGCC-3′, 5′-CCATCTGAAAGTTTTGCAAG-3′, *T*_*m*_ = 60°C, 1442 bp.

### 2.2. Analysis of mRNA, Semiquantitative RT-PCR, and Protein Expression

Total RNA was isolated from heart homogenates with a TRIzol reagent (Invitrogen, Carlsbad, CA). Genomic DNA contamination in the RNA samples was removed through DNase I digestion, and cDNA of the first strand was synthesized using ReverTra Ace reverse transcriptase (Toyobo, Japan) and oligo(dT) as the primer. The following PCR primers were used for amplifying the target genes for the results in Figure 1(b)(B): Human HSP60: the same as listed in (I); human and mouse HSP60: 5′-GTCAGAAATGTGAATTCCAG-3′, 5′-TTGACTGCCACAACCTGAAGAC-3′, *T*_*m*_ = 55°C, 200 bp; and GAPDH: 5′-GTGGCAAAGTGGAGATTGCC-3′, 5′-GATGATGACCCGTTTGGCTCC-3′, *T*_*m*_ = 58°C, 290 bp. Ten micrograms of total proteins isolated from tissue homogenates was subjected to 10% SDS-PAGE, followed by Western blotting using anti-HSP60 (ab5479, Abcam) and anti-GFP (ab290) antibodies. Ten micrograms of total proteins isolated from MDA-MB231 cells was used for comparison.

### 2.3. Fluorescence Detection and Histological Analysis

Hearts of neonatal mice and E17 mouse embryos were washed with PBS and fixed by 10% neutral formalin. Stereo microscope (Olympus SZX10) with fluorescence module was used for observing green fluorescence of neonatal mouse hearts. For investigating septum defects, unfixed neonatal hearts were immersed in OCT matrix (Tissue-Tek, USA) and frozen quickly in liquid nitrogen. The samples were cryosectioned 10 *μ*m thick followed by hematoxylin and eosin (H&E) staining to reveal the four-chamber view of hearts. In parallel, formalin-fixed hearts were embedded in paraffin and cut into serial 5 *μ*m sections and stained with H&E to evaluate morphology.

### 2.4. Immunohistochemical and Immunofluorescence Assay

Paraffin-embedded tissue sections were dewaxed, rehydrated, and treated by 3% hydrogen peroxide in methanol for 30 min. Tissue sections were then incubated in blocking solution (5% normal goat serum and 0.3% Triton-X 100 in PBS) followed by incubating with HSP60 (SC-1052, Santa Cruz, CA) overnight at 4°C. The sections were rinsed and incubated with biotinylated donkey anti-goat immunoglobulin G (IgG) and then reacted to Vector Elite ABC in which the color was developed with diaminobenzidine. Images were taken by using the Olympus DP72 CCD attached to an Olympus BX51 microscope with DP controller. For the immunofluorescence staining, antibodies of cleaved caspase-3 (Cell Signaling 9961), CD4 (MAB554), and CD8*α* (MAB116) were used for incubating samples overnight in blocking solution at 4°C. Alexa Fluor 488 conjugated goat anti-rabbit IgG (H+L) or goat anti-mouse IgG (H+L) secondary antibodies (Invitrogen) were used instead. The mounting media containing DAPI (Invitrogen) were used for counterstaining cell nucleus.

### 2.5. TUNEL Assay

Terminal deoxynucleotidyl transferase-mediated dUTP nick-end labeling (TUNEL) assay was performed for detecting nuclear DNA fragmentation in paraffin-embedded tissue sections as a measure of apoptosis. Fluorescein-12-dUTP was used as the substrate in TUNEL reaction (DeadEnd Fluorometric TUNEL System, Promega); the slides were counterstained by DAPI, and the images were taken by the Olympus microscope system.

### 2.6. T2-Weighted Thoracic Region MR Images

Magnetic resonance imaging (MRI) was performed and images were acquired using 9.4 Tesla MRI system (Bruker, Ettlingen, Germany) with a maximum gradient strength of 140 G cm^−1^. A quadrature volume coil (internal diameter = 10 mm) was used for RF transmission and reception. We used a rapid acquisition with relaxation enhancement sequence to obtain the T2-weighted images. The repetition and echo times were set at 2500 and 3.4 milliseconds, respectively. The field of view was 10 × 10 mm^2^ and the matrix size was 100 × 100. The images were sliced without gaps, and the slice thickness was 0.2 mm. The voxel size for acquisition was 0.1 × 0.1 × 0.2 mm^3^. Finally, the images were interpolated to 400 × 400 using cubic interpolation.

### 2.7. Electron Micrograph

Neonatal heart tissues were collected and fixed in 4% cold glutaraldehyde, postfixed using 1% osmium oxide, and progressively dehydrated using alcohol. After resin embedding and polymerization, 1 *μ*m thick sections were cut for initial observation. Ultrathin (60–80 nm thick) sections were cut from the Epon-embedded blocks, stained with uranyl acetate and lead citrate, and examined using transmission electron microscopy (TEM, Hitachi H-7500, Tokyo, Japan) at 75 kV.

## 3. Results and Discussion

### 3.1. Generation of HSP60 Conditional Transgenic Mice

**Figure 2:**
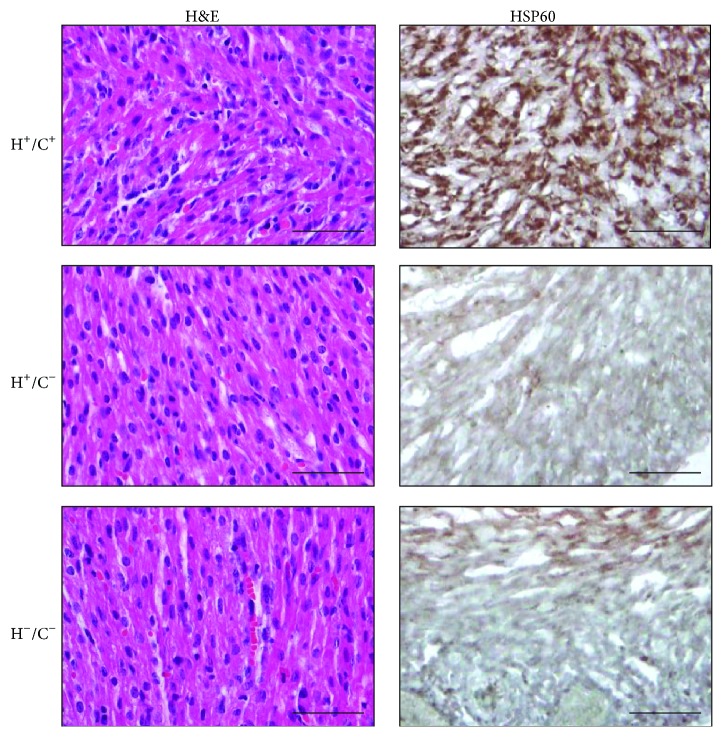
HSP60 detection in neonatal H^+^/C^+^ mice heart. H&E and immunohistochemical staining of HSP60 induction in H^+^/C^+^ heart sections, but not in H^+^/C^−^ or H^−^/C^−^ heart sections. Scale bar = 50 *μ*m.

Unlike Tg mice expressing HSP10 [35], HSP20 [36], HSP27 [37], or HSP70 [38], founders for establishing the ubiquitous HSP60 Tg mouse were not viable. In this study, we generated a conditional Tg mouse model using Cre-LoxP tools to allow inducible and tissue-specific HSP60 expression. G-Lox-HSP60 mouse was generated by using the Tg vector illustrated in Figure 1(a). CAGGS was used to drive the expression of transgenes, enhanced green fluorescent protein (EGFP), or human HSP60 full-length cDNA. Multiple stop codons and the SV40 polyA sequences between the 2 LoxP sites of the Tg vector prevent the expression of downstream human HSP60 sequence. The G-Lox-HSP60 mice were normal in development, weight, and reproduction, with EGFP expression in most organs. During Cre-mediated recombination, 2 LoxP sites of the G-Lox-HSP60 vector are joined, and the HSP60 expression is directly enhanced by CAGGS. In the present study, we generated an FVB ubiquitous Cre Tg mouse expressing the EGFP-Cre fusion protein [33]. We used the tail DNA-PCR method for studying the genotypes of the littermates in the crossing studies of G-Lox-HSP60 and EGFP-Cre mice; the results are described in Figure 1(b). Deletion of the LoxP-flanked cassette was detected by the presence of the shorter PCR fragment in the double Tg mice. Littermates with all 4 possible genotypes were acquired in accordance with the Mendelian frequency, indicating no prenatal loss of the double Tg mice (H^+^/C^+^), which were positive for both G-Lox-HSP60 and EGFP-Cre alleles (Table 1). Strong induction of human HSP60 expression in the cardiac tissues of the double Tg mice was confirmed through RT-PCR, Western blotting (Figure 1(b)), and IHC staining (Figure 2).

### 3.2. HSP60 Expression Led to Neonatal Deaths

**Figure 3:**
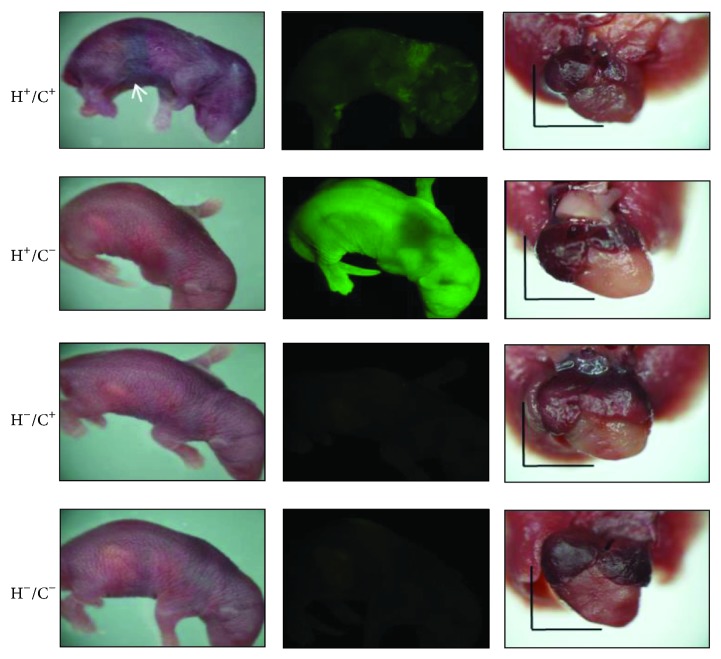
Neonatal Tg mice. Left, neonatal Tg mice. The white arrow indicates cyanosis and abdominal bleeding in H^+^/C^+^ neonates; middle, fluorescent images of the same mice; right, the lungs and heart of neonatal mice. H^+^/C^+^ hearts appear more dark reddish and smaller than hearts of other genotypes. Scale bar = 3 mm.

In more than 20 crossing experiments using G-Lox-HSP60 and EGFP-Cre mice, all double Tg HSP60 mice litters died within a few hours after birth. A few survived the first day but none survived more than a few days (72/72, Table 1). Neonatal deaths were rare in single Tg mice—H^+^/C^−^ (5/76) and H^−^/C^+^ (2/69)—which carried only G-Lox-HSP60 or EGFP-Cre, and in the wild-type mice (H^−^/C^−^, 2/86). At birth, neonatal HSP60 mice were slightly smaller in size than average, with signs of mild cyanosis and internal bleeding at the abdominal segments before death (Figure 3). Severe cardiovascular or pulmonary abnormality was suspected to contribute to the neonatal deaths in the HSP60 mice. H^+^/C^−^ mice exhibited strong whole-body green fluorescence, whereas the HSP60 mice displayed green fluorescent spots, indicating remaining EGFP that was produced before recombination. H^−^/C^+^ and H^−^/C^−^ mice did not exhibit fluorescence. Thoracic chamber dissection of HSP60 mice revealed a smaller heart, and the color of the ventricles was more reddish, containing more blood than the hearts of other genotypes (Figure 3).

**Figure 4:**
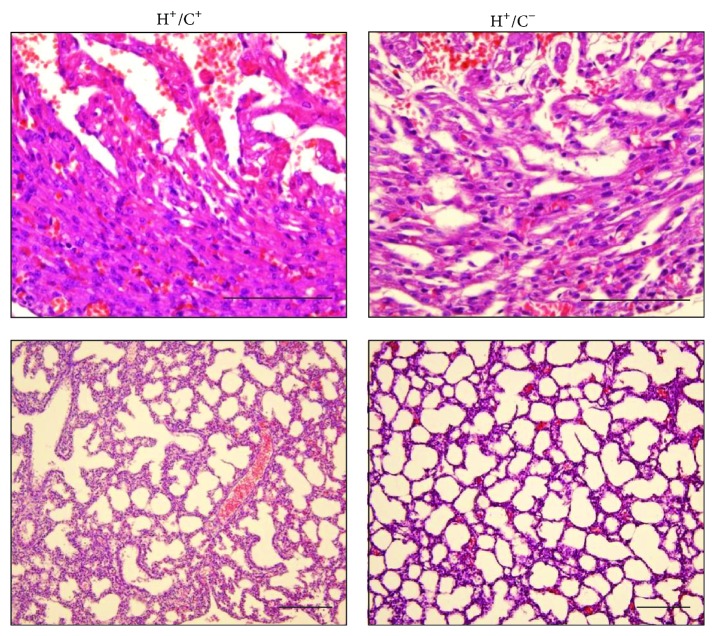
Histology of the neonatal heart and lung. Top, H&E staining of the neonatal heart of H^+^/C^+^ mouse compared with that of H^+^/C^−^ mouse, showing hemorrhage, necrosis, and degenerated cardiac muscles in H^+^/C^+^ mouse heart. Bottom, the lung of H^+^/C^+^ mouse shows signs of hemorrhage, liquid congestion, and tissue necrosis. The alveolar lung structures appeared to have developed normally but poorly inflated. Scale bar = 100 *μ*m.

**Figure 5:**
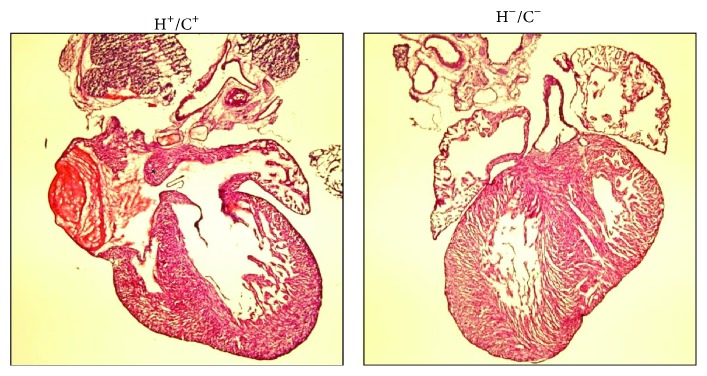
Atrial septal defect in H^+^/C^+^ neonatal mice. H&E images of the neonatal heart from H^+^/C^+^ and H^−^/C^−^ mice. Asterisk indicates the atrial septal defect.

Postmortem analysis of the HSP60 mice was performed through MRI and histology. MRI revealed that the HSP60 P1 mouse heart was smaller than that of H^+^/C^−^ mouse (Figure S1 in Supplementary Material available online at http://dx.doi.org/10.1155/2015/539805). The HSP60 mouse heart chambers and lungs were filled with liquids that were absent in those of H^+^/C^−^ mouse. The interventricular septum and chamber walls of the HSP60 mice were thinner than normal (Figure S1). Histological examination revealed massive hemorrhage at the heart and lungs of double Tg mouse. The cardiac muscle appeared degenerated or incompletely differentiated, with sponge-like cardiac tissues fenestrated with blood or blood vessels, and signs of myocardium necrosis were observed (Figure 4). The lungs of the HSP60 mice appeared to have developed normally except for the presence of hemorrhage that potentially resulted in only partial inflation of the lungs. Other organs, such as the brain, kidneys, and liver, of the HSP60 mice appeared grossly normal (data not shown). Spotty hemorrhage was observed in the skeletal muscles of the neck, spine, and between the ribs; however, the hemorrhage in these organs was not as severe as that in the heart and lungs. Because cardiovascular and respiratory stresses were observed and because the hearts were smaller than normal, we speculate that congenital defects may contribute to the neonatal deaths of the HSP60 mice. Of the 10 HSP60 mice studied for septal defects, 4 had atrial septal defect (ASD) and one had ventricular septal defect (VSD), as illustrated in Figure 5. Septum defects were not observed in the control mice.

Because the atrial septum is closed when the lungs are inflated during birthing and because ASD manifests as a serious problem only after birth, we examined whether the HSP60 mice had developmental defects during the embryonic stage in the E17 embryos. No apparent abnormalities were visible externally in the HSP60 mouse E17 embryos. However, the embryo sections revealed excessive hemorrhage, vascularization, and tissue necrosis in the heart, similar to those in the postnatal heart. Lungs of E17 of HSP60 mouse appeared grossly normal (Figure S2). Thus, histological evidence suggested that congenital heart disease caused by cardiomyopathy or incomplete heart development in HSP60 mouse embryos results in ASD or VSD, which aggravates respiratory stress and congestive heart failure at postnatal day 1.

**Figure 6:**
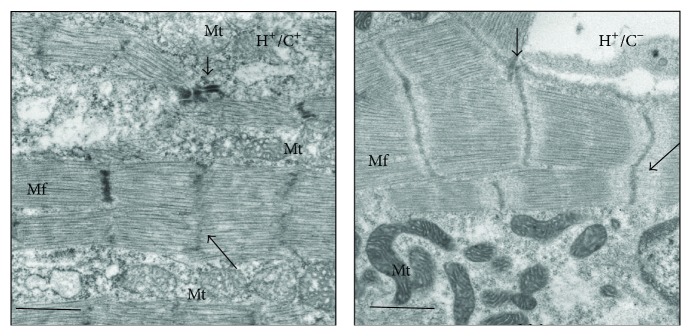
Transmission electron microscopy showing ultrastructure of myofibril defect in H^+^/C^+^ neonatal heart. Transmission electron microscopy results of H^+^/C^+^ (left) and H^+^/C^−^ (right) mice left ventricular tissue. Mf, myofibrils; Mt, mitochondria; long arrow, Z disk. Original magnification 10,000x; scale bar = 1 *μ*m.

We searched for ultrastructural abnormality as features of cardiac myopathy in neonatal HSP60 mouse using electron micrographs. G-Lox-HSP60 mouse contained organized arrays of myofibril filaments with intact mitochondrion appearing in high contrast with dense laminae cristae throughout the mitochondrial cross-sectional areas (Figure 6). In contrast, the myofibrils of HSP60 mouse heart exhibited pronounced fragmentation and disorganization, shown as ragged Z lines, with shorter sarcomere length on average compared with G-Lox-HSP60 myofibrils. Mitochondrion between myofibrils was mostly broken, losing both outline and inner cristae, indicating a disintegrated mitochondrial structure. Thus, TEM results confirmed severe cardiac myopathy in HSP60 mouse.

### 3.3. Increasing Apoptosis in HSP60 Mouse Heart

**Figure 7:**
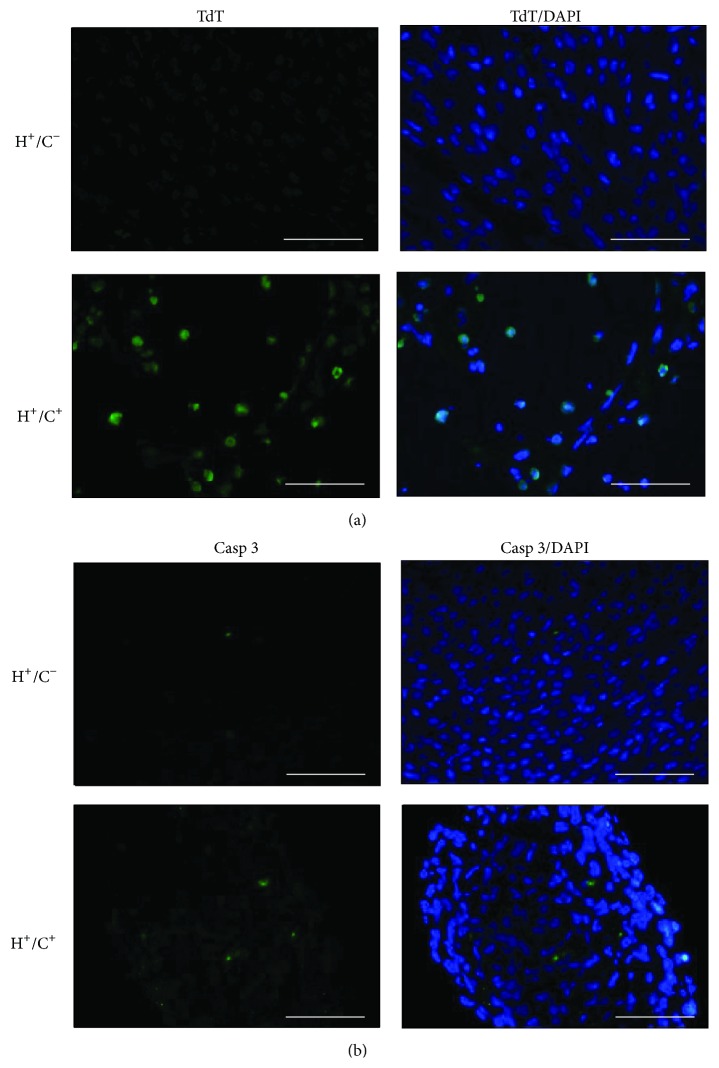
TUNEL staining and activated caspase-3 detection in neonatal heart sections. (a) Detection of nuclear DNA fragmentation with TUNEL reaction in the left ventricle sections of neonatal H^+^/C^+^ and H^+^/C^−^ mice. Fluorescein-12-dUTP (TdT) and DAPI were used for TUNEL and nuclear staining. (b) Immunofluorescence detection of activated (cleaved) caspase-3 induction in H^+^/C^+^ and in H^+^/C^−^ heart sections. Scale bar = 50 *μ*m.

HSP60 was previously linked to apoptosis induction; thus, we studied apoptotic cardiac myocytes in the HSP60 mouse by using the TUNEL assay. Many cells in the HSP60 mouse heart sections were positively labeled by fluorescein-dUTP at the cell nucleus through TUNEL assay, whereas very few TUNEL positively cells were in H^+^/C^−^ mouse heart sections (Figure 7(a)). In parallel, a few cells in the HSP60 mouse heart section were positively stained by anti-activated caspase-3 antibody, significantly more than that of H^+^/C^−^ mouse heart (Figure 7(b)). The data clearly demonstrated that HSP60 overexpression results in caspase-3 activation and apoptosis of myocardial cells in HSP60 mice. Because the HSP60 mouse heart became a sponge-like muscular tissue infiltrated by small capillaries and HSP60 involvement in rejection and autoimmune diseases was suggested previously, we studied the presence of activated inflammatory cells in HSP60 mice. The pathology neither showed infiltrated mononuclear cells (Figure 4) nor detected CD4 or CD8 positive cells in HSP60 mouse heart (Figure S3); thus inflammatory response or T cell activation is absent in the HSP60 mouse heart.

Accumulation of HSP60 in the cytoplasm during apoptosis induction and the binding of HSP60 to procaspase-3 and modulating caspase activity has been demonstrated in many cancer cell lines [21, 25, 39]. Similar to most proteins of the mitochondrial matrix, HSP60, which is synthesized in the cytoplasm with N-terminal targeting peptides, is transported to the mitochondrial matrix, where the targeting sequence is cleaved. Both unprocessed (cytosolic) and matured (mitochondrial) HSP60 strongly increased in the cardiac tissue of HSP60 mouse (Figure 1(b)). Consequently, accumulation of cytosolic HSP60, capable of inducing apoptosis, cannot be overlooked and our data provided* in vivo* evidence of apoptosis and caspase-3 activation in the HSP60 mouse heart (Figure 7). Apoptosis is a highly regulated process during embryonic and postnatal heart development and critical for heart development. Increasing apoptosis in HSP60 mouse heart and its causal relation to cardiac myopathy must be interpreted with caution because (1) apoptosis frequently occurs in embryonic and neonatal tissues than in adult cardiac tissues; (2) increasing apoptosis is often observed in cardiac tissues of myopathy and congestive heart failure; thus apoptosis can be secondarily induced in myopathy and heart failure, and (3) it can directly result from cytosolic HSP60 accumulation or HSP60 binding to caspases. Thus, additional studies are required in this regard. Our current model, which uses the ubiquitous Cre mouse, increased HSP60 and potentially induced cell apoptosis of multiple tissues; thus it is unable to clarify phenotypes contributed by the HSP60 induction in a specific organ, for example, vascular ECs, smooth muscle cells, or cardiac myocytes. This limitation can be overcome by combinatory uses of G-Lox-HSP60 with vascular- or cardiac-specific Cre mice. We have explored HSP60 expression in cardiac tissues of the adult mice by using inducible cardiac cell-restricted Cre mouse (unpublished results).

In addition to the roles of mitochondrial and cytosolic chaperonins and caspase induction, additional functions of HSP60 have been reported. Cytosolic HSP60 regulates the phosphorylation of IKK*α*/*β* and promotes NF-*κ*B prosurvival pathway. Transgenic expression of the cytosolic HSP60 prevented stress-induced hepatic cell deaths* in vivo* [32]. Overexpressing HSP60 in the human stem cells increased HSP60 nuclear localization, which suppressed ROS and p38/JNK signaling and regulated genes relating to stem cell proliferation, differentiation, and stemness [40]. Whether the aforementioned mechanisms are also involved in mediating neonatal deaths remains unclear, and future clarification is awaited.

In this study, congenital ASD was observed in a large number of HSP60 mice. Although ASD exacerbated the existing myopathy of HSP60 mice and was partially responsible for neonatal death, ASD* per se* was unlikely directly caused by HSP60 induction. We speculate that ASD in neonatal HSP60 mice may be the consequence of incomplete embryonic development, existing myopathy, and increasing apoptosis. Similar phenomenon has been demonstrated in the eNOS knockout mouse model, in which high incidence of ASD or VSD was precipitated through increased apoptosis during embryonic and postnatal stages [41]. Our main findings indicate that severe hemorrhage and myocyte death occurred in mice with HSP60 expression, and increased apoptosis in a macerated and spongy-like heart failed to meet the cardiovascular demands after birth and was the primary cause of HSP60-induced heart failure and neonatal death.

## 4. Conclusions

HSP60 not only is the essential mitochondrial chaperone protein but also plays critical roles outside the mitochondrion. Although numerous studies indicated the wide involvement of HSP60 in broad biological processes and many diseases, reports of transgenic mouse models of increasing or knockdown HSP60 expression are rare. Thus, the consequences of altering HSP60 levels* in vivo* in these disease settings remain unknown. We used a floxed vector to establish conditional HSP60 transgenic mice, which allows studies on inducible and organ-targeted HSP60 expression in adult mice and is potentially useful in pursuing essential questions related to HSP60* in vivo*. In this study, we show that ubiquitous HSP60 expression from the embryonic stage results in increased apoptosis, myopathy, high incidence of ASD, and neonatal deaths.

## Supplementary Material

Results supplement to Chen et al. Neonatal Death and Heart Failure in Mouse with Transgenic HSP60 Expression.
Figure S1. T2-weighted MR images of the thoracic region of H^+^/C^+^ and H^+^/C^−^ neonatal mice
Images of the H^+^/C^+^ heart (left) and H^+^/C^−^ heart (right) from atrium (top) toward ventricle (bottom). Slices 1 (top), 4, 7, 10, and 13 (bottom) with slice thickness of 0.2 mm are shown. SC, spinal cord; Ln, Lungs; LRA, lumen of the right atrium; LLA, lumen of the left atrium; LRV, lumen of the right ventricle; LLV, lumen of the left ventricle; WRV, wall of the right ventricle; WLV, wall of the left ventricle; IVS, interventricular septum; LVC: left cranial vena cava.
Figure S2. Hemorrhage and myopathy in E17 H^+^/C^+^ embryos
A: H&E images of the cardiac tissue from H^+^/C^+^ (a, b, c) and H^+^/C− (d) E17 embryos. a. H^+^/C^+^ mouse showing hemorrhage and sponge-like muscular tissue with dense eosin staining. b. Enlarged picture of intraventricular septum (at a. white arrowhead) showing hemorrhage, necrosis, and smaller cytoplasmic volume. c. Free wall of LV (at a. black arrowhead). The LV wall was thin, formed by only a few layers of cells; in some area, the wall was completely eroded by blood vessels (black arrow). d. The intraventricular septum of the H^+^/C^−^ E17 embryo heart showing normal nucleus and myocytes. Scale bars in b, c, d = 25 μm.
B: Lungs of H^+^/C^+^ (a) and H^+^/C^−^ (b) mouse embryo. E17 H^+^/C^+^ lung displayed patched areas of hemorrhage and necrosis amid otherwise normal in development. Scale bar = 100 μm.
Figure S3. Activation of CD4 or CD8 T cells was not observed in neonatal H^+^/C^+^ heart
Absence of CD4 or CD8 positive cells in neonatal H^+^/C^+^ hearts (A, C) or in H^+^/C^−^ heart (B, D). Anti-CD4 (A, B) or anti-CD8α (C, D) antibodies were used. DAPI was used to counter stain the nuclei. Scale bar = 50 μm.

## Figures and Tables

**Table 1:** Summary of crossings between G-Lox-HSP60 (H) and EGFP-Cre (C) mice.

Genotype	H^+^/C^+^	H^+^/C^−^	H^−^/C^+^	H^−^/C^−^	Total
Newborn number	72	76	69	86	303
Neonatal death number	72	5	2	2	81

Ratio	72/72	5/76	2/69	2/86	81/303

H^+^/C^+^, double Tg mouse; H^+^/C^−^, H^−^/C^+^, single Tg mouse carrying either allele, H^−^/C^−^, wild-type mouse.

## Abbreviation

ATP: Adenosine triphosphate
TNF-*α*: Tumor necrosis factor-*α*
IL-1*β*: Interleukin 1*β*
IL-6: Interleukin 6
NO: Nitric oxide
NOD: Nonobese diabetic
ASD: Atrial septum defect
VSD: Ventricular septum defect
ROS: Reactive oxygen species
eNOS: Endothelial nitric oxide synthase
CAGGS: CMV enhancer/chicken *β*-actin
H&E: Hematoxylin and eosin.
