# Supplementary figures and images for: Neonatal Death and Heart Failure in Mouse with Transgenic HSP60 Expression
Source: Biomed Res Int. 2015 Oct 4;2015:539805. doi: 10.1155/2015/539805 (PMC4609373; doi:10.1155/2015/539805)

Figure S1

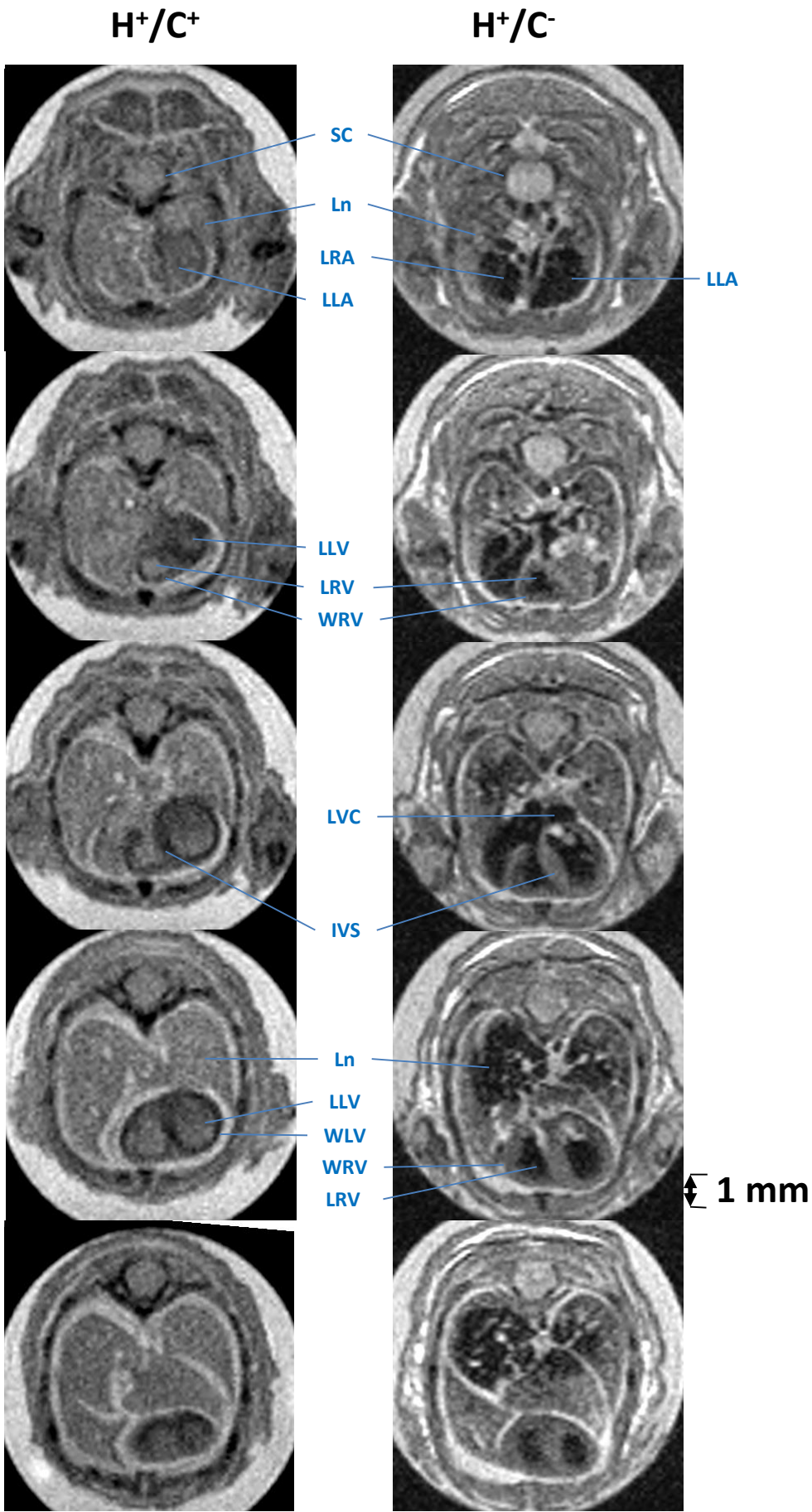

**Figure S2**

**A**

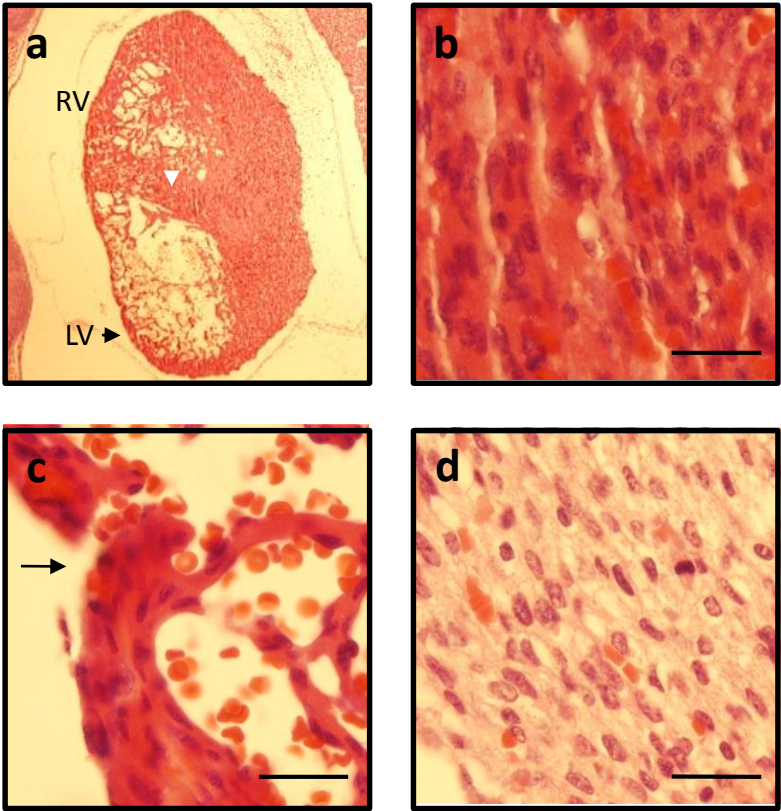

**B**

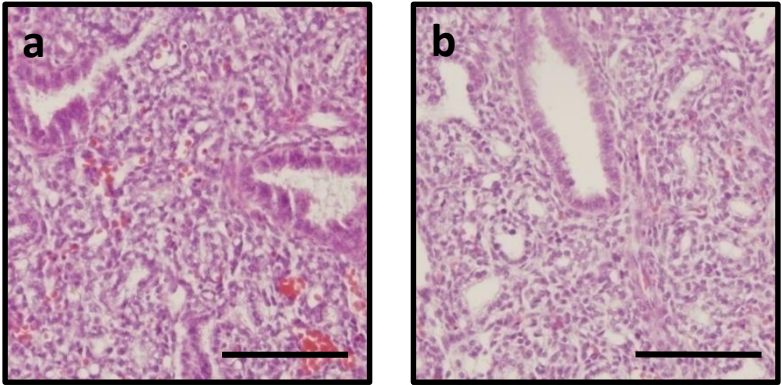

**Figure S3**

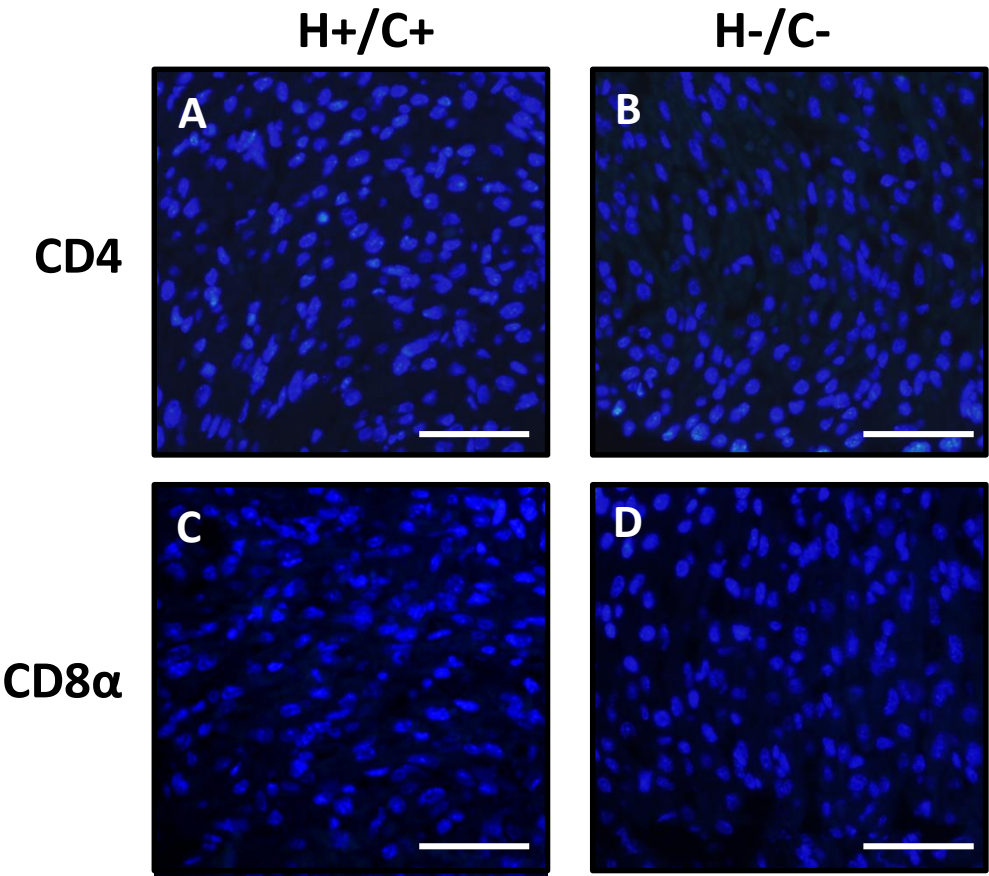

Supplement: Supplementary file 1 — Results supplement to Chen et al. Neonatal Death and Heart Failure in Mouse with Transgenic HSP60 Expression. Figure S1. T2-weighted MR images of the thoracic region of H+/C+ and H+/C− neonatal mice Images of the H+/C+ heart (left) and H+/C− heart (right) from atrium (top) toward ventricle (bottom). Slices 1 (top), 4, 7, 10, and 13 (bottom) with slice thickness of 0.2 mm are shown. SC, spinal cord; Ln, Lungs; LRA, lumen of the right atrium; LLA, lumen of the left atrium; LRV, lumen of the right ventricle; LLV, lumen of the left ventricle; WRV, wall of the right ventricle; WLV, wall of the left ventricle; IVS, interventricular septum; LVC: left cranial vena cava. Figure S2. Hemorrhage and myopathy in E17 H+/C+ embryos A: H&E images of the cardiac tissue from H+/C+ (a, b, c) and H+/C− (d) E17 embryos. a. H+/C+ mouse showing hemorrhage and sponge-like muscular tissue with dense eosin staining. b. Enlarged picture of intraventricular septum (at a. white arrowhead) showing hemorrhage, necrosis, and smaller cytoplasmic volume. c. Free wall of LV (at a. black arrowhead). The LV wall was thin, formed by only a few layers of cells; in some area, the wall was completely eroded by blood vessels (black arrow). d. The intraventricular septum of the H+/C− E17 embryo heart showing normal nucleus and myocytes. Scale bars in b, c, d = 25 μm. B: Lungs of H+/C+ (a) and H+/C− (b) mouse embryo. E17 H+/C+ lung displayed patched areas of hemorrhage and necrosis amid otherwise normal in development. Scale bar = 100 μm. Figure S3. Activation of CD4 or CD8 T cells was not observed in neonatal H+/C+ heart Absence of CD4 or CD8 positive cells in neonatal H+/C+ hearts (A, C) or in H+/C− heart (B, D). Anti-CD4 (A, B) or anti-CD8α (C, D) antibodies were used. DAPI was used to counter stain the nuclei. Scale bar = 50 μm. [file 539805.f1.pdf]
